# P-765. Enhancing Tuberculosis (TB) Surveillance with Artificial Intelligence: Utilizing Latent Semantic Indexing (LSI) to Automatically Detect TB Cases

**DOI:** 10.1093/ofid/ofae631.960

**Published:** 2025-01-29

**Authors:** Bráulio R G M Couto, Marco Aurélio Angelo, Débora De Vasconcelos, Giovana Ferreira, Meritxell Bassas, Laiz Almeida, Rossana Souza, Walisson Ferreira Carvalho, Naísses Zóia Lima, Ana Paula Ladeira

**Affiliations:** AMECI – Associação Mineira de Epidemiologia e Controle de Infecções, Belo Horizonte, Minas Gerais, Brazil; Hospital Risoleta Tolentino Neves - HRTN, Belo Horizonte, Minas Gerais, Brazil; Hospital Risoleta Tolentino Neves - HRTN, Belo Horizonte, Minas Gerais, Brazil; Hospital Risoleta Tolentino Neves - HRTN, Belo Horizonte, Minas Gerais, Brazil; Hospital Risoleta Tolentino Neves - HRTN, Belo Horizonte, Minas Gerais, Brazil; Hospital Risoleta Tolentino Neves - HRTN, Belo Horizonte, Minas Gerais, Brazil; Biobyte Sistemas, Belo Horizonte, Minas Gerais, Brazil; PUC MInas, Belo Horizonte, Minas Gerais, Brazil; PUC MInas, Belo Horizonte, Minas Gerais, Brazil; Biobyte Tecnologia em Epidemiologia, Belo Horizonte, Minas Gerais, Brazil

## Abstract

**Background:**

Reducing TB surveillance incurs significant costs, as effective control and societal impact minimization rely on robust, ongoing surveillance systems that integrate and analyze comprehensive data for prompt action. Our study supports integrating artificial intelligence (LSI) into TB and other notifiable disease monitoring frameworks to improve tracking

**Figure 1:**
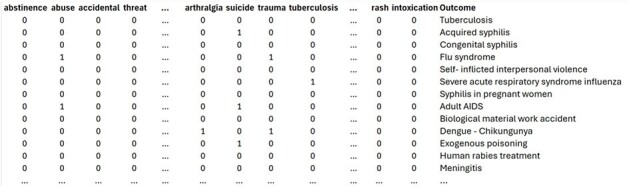
Binary Matrix of 125 Terms: Presence vs. Absence of Root Keywords Retrieved from the Patient Electronic Record for the LSI Automatic Retrieval System. Binary Matrix of 125 Terms: Presence vs. Absence of Root Keywords Retrieved from the Patient Electronic Record for the LSI Automatic Retrieval System.

**Methods:**

In 2024, we developed a novel application within Datamart A.R.G.U.S.—Assistant for Recovery and Guarding of Urgent and Epidemiological Sentinels—a cloud-based platform hosted on AWS and developed by our team. This application integrates with hospital systems (MV) and laboratory systems (MATRIX and GAL) to enhance the surveillance of healthcare-associated infections (HAI) and notifiable diseases. Data from both hospital and outpatient care are encoded into a binary matrix of 125 terms (root keywords) extracted from patient electronic records. This matrix is then processed using Singular Value Decomposition (SVD) within an LSI-based information retrieval system (Fig. 1). In our model, each patient is represented as a point in a 125-dimensional hyperdimensional space. When projected into three-dimensional space, this framework allows for effective clustering and classification of notifiable Diseases (Fig. 2), specifically TB (Fig. 3).

**Figure 2:**
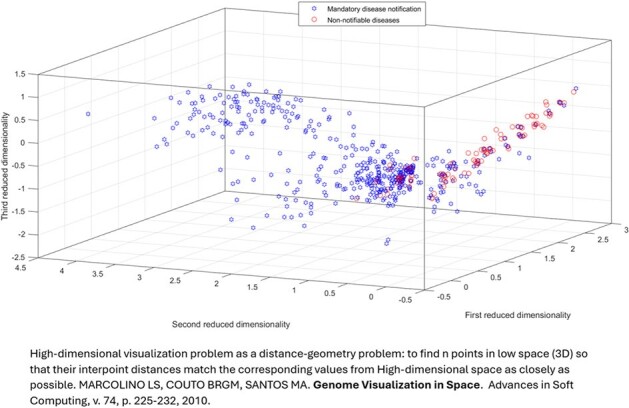
3D Visualization of High-Dimensional Data: Comparing Mandatory Notifiable Diseases versus Non-Notifiable Diseases. Both patient types are well separated in the space, enabling automatic clustering and classification. 3D Visualization of High-Dimensional Data: Comparing Mandatory Notifiable Diseases versus Non-Notifiable Diseases. Both patient types are well separated in the space, enabling automatic clustering and classification.

**Results:**

The LSI-based matrix was constructed using data from January 2023 to February 2024, encompassing a sample of 17,895 cases (lines) across 125 presence versus absence terms, with each case classified as either a notifiable or non-notifiable disease. The 125-dimensional matrix was reduced to 28 factors using SVD. The sensitivity and specificity of the Euclidean distance were better than those of cosine similarity when automatically identifying TB cases in 1,970 independent queries (Fig. 4).

**Figure 3:**
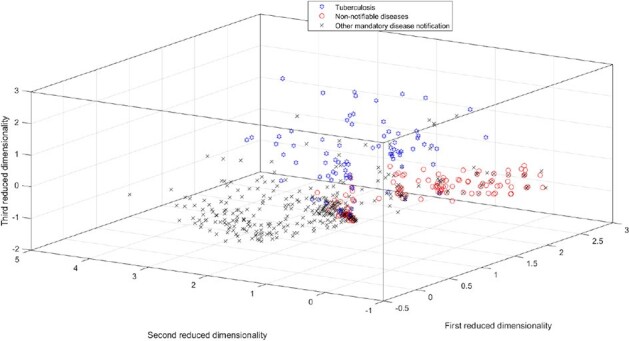
3D Visualization of High-Dimensional Data: Comparing Tuberculosis versus Other Mandatory Notifiable Diseases versus Non-Notifiable Diseases. The three types of patients are well separated in the space, enabling automatic clustering and classification. 3D Visualization of High-Dimensional Data: Comparing Tuberculosis versus Other Mandatory Notifiable Diseases versus Non-Notifiable Diseases. The three types of patients are well separated in the space, enabling automatic clustering and classification.

**Conclusion:**

Artificial Intelligence (LSI), significantly enhances the surveillance of TB and other notifiable diseases by enabling real-time, automated case identification up to one day prior (D-1). This advancement allows surveillance personnel to complete their tasks more efficiently, thereby preventing outbreaks and reducing the risk of unforeseen fatalities.

**Figure 4:**
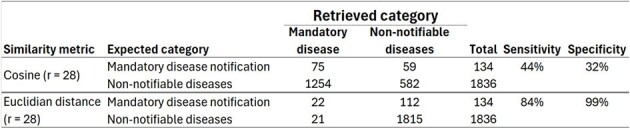
Performance Comparison of Cosine vs. Euclidean Distance Metrics in Automatically Identifying TB Cases Using LSI: Euclidean Distance Demonstrates Superior Accuracy and Has Been Integrated into Datamart A.R.G.U.S. for Real-Time Identification of TB and Other Notifiable Diseases as of the Previous Workday (D-1). Performance Comparison of Cosine vs. Euclidean Distance Metrics in Automatically Identifying TB Cases Using LSI: Euclidean Distance Demonstrates Superior Accuracy and Has Been Integrated into Datamart A.R.G.U.S. for Real-Time Identification of TB and Other Notifiable Diseases as of the Previous Workday (D-1).

**Disclosures:**

**All Authors**: No reported disclosures

